# Connexins, Pannexins and Gap Junctions in Perinatal Brain Injury

**DOI:** 10.3390/biomedicines10061445

**Published:** 2022-06-18

**Authors:** Alice McDouall, Kelly Q. Zhou, Laura Bennet, Colin R. Green, Alistair J. Gunn, Joanne O. Davidson

**Affiliations:** 1 U1 Department of Physiology, Faculty of Medical and Health Sciences, The University of Auckland, Auckland 1023, New Zealand; a.mcdouall@auckland.ac.nz (A.M.); k.zhou@auckland.ac.nz (K.Q.Z.); l.bennet@auckland.ac.nz (L.B.); aj.gunn@auckland.ac.nz (A.J.G.); 2 Department of Ophthalmology, Faculty of Medical and Health Sciences, The University of Auckland, Auckland 1023, New Zealand; c.green@auckland.ac.nz

**Keywords:** connexin, pannexin, gap junction, hemichannel, ischemia, inflammation, inflammasome, ATP

## Abstract

Perinatal brain injury secondary to hypoxia-ischemia and/or infection/inflammation remains a major cause of disability. Therapeutic hypothermia significantly improves outcomes, but in randomized controlled trials nearly half of infants still died or survived with disability, showing that additional interventions are needed. There is growing evidence that brain injury spreads over time from injured to previously uninjured regions of the brain. At least in part, this spread is related to opening of connexin hemichannels and pannexin channels, both of which are large conductance membrane channels found in many brain cells. Opening of these membrane channels releases adenosine triphosphate (ATP), and other neuroactive molecules, into the extracellular space. ATP has an important role in normal signaling, but pathologically can trigger the assembly of the multi-protein inflammasome complex. The inflammasome complex promotes activation of inflammatory caspases, and release of inflammatory cytokines. Overall, the connexin hemichannel appears to play a primary role in propagation of injury and chronic disease, and connexin hemichannel blockade has been shown to be neuroprotective in multiple animal models. Thus, there is potential for some blockers of connexin or pannexin channels to be developed into targeted interventions that could be used in conjunction with or separate to therapeutic hypothermia.

## 1. Introduction

Perinatal brain injury is associated with increased risk of mortality and long-term neurodevelopmental impairments at all gestational ages. In term infants, the most common cause of perinatal brain injury is hypoxia ischemia (HI). Moderate to severe hypoxic-ischemic encephalopathy (HIE) occurs in 1–3/1000 live births in high resource settings [1,2]. Moderate to severe HIE is substantially higher in lower resource settings, with for example, an estimated incidence of 13/1000 live births in Uganda [3] and 52/1000 live births in Tanzania [4].

In preterm infants, small retrospective studies suggested that the incidence of moderate to severe HIE is 1–8/1000 live births [5,6,7]. However, a large cohort study in the United States of America suggested that this could be much higher with a rate of 37.3/1000 live births before 37 weeks of gestation [8]. Perinatal brain injury in preterm infants can also be caused by infection, including infection of the fetal membranes (chorioamnionitis), which is linked to between 11% and 40% of preterm births [9]. Furthermore, prematurity itself makes the infant more susceptible to abnormal outcomes as proliferation and maturation have not been completed. It is likely that interactions between HI, infection, and prematurity all together result in the development of perinatal brain injury.

Currently there is only one proven neuroprotective treatment for term infants with moderate to severe HIE, namely, therapeutic hypothermia (TH). TH involves cooling either the head to 34.5 ± 0.5 °C or the whole body to 33.5 ± 0.5 °C, started as early as possible in the first 6 h after birth and then continued for 72 h [10]. TH significantly reduces mortality, brain injury, and major neurodevelopmental disability [11,12,13,14]. However, TH is only partially effective, with a number needed to treat of eight, and in the large randomized controlled trials (RCTs) nearly half of infants treated with TH had adverse outcomes [10]. Currently, TH is not recommended for term infants with mild HIE because this group was not included in the original RCTs, albeit there has been considerable therapeutic drift in many centers, as reviewed in [15]. TH is not recommended for preterm infants because of evidence that mild hypothermia is associated with increased mortality [16,17].

TH has some limitations including that treatment typically requires that the infant should be transferred to a specialist neonatal intensive care unit (NICU), will often require sedation, separation from the parents and, in many cases, delayed oral feeding. Although no serious side effects of TH have been reported, it is consistently associated with mild bradycardia, and an overall mild fall in thrombocyte counts [10]. Critically, TH is not established for use outside of high-income countries. The hypothermia for neonatal encephalopathy in low- and middle-income countries (HELIX) trial, a multi-center, multi-country RCT, found that TH did not reduce the combined outcome of death or disability at 18 months and indeed, that TH was associated with increased mortality [18]. Consequently, there is significant interest in the development of neuroprotective strategies that would be complementary with TH or used as sole therapy for perinatal encephalopathy [19,20].

A striking feature of many forms of HI is that injury evolves over time and can spread from severely injured areas into parts of brain that were initially intact [21,22]. This evolution of brain injury after HI can be broadly characterized into four phases; the primary, latent, secondary, and tertiary phase [23]. The primary phase of injury occurs during HI, when oxidative metabolism collapses, resulting in cellular depolarization, edema, and necrosis [19], as well as extracellular accumulation of excitatory amino acids [24]. After blood and oxygen supply is restored, the latent phase begins, in which there is a transient restoration of oxidative metabolism [23]. It is during the latent phase that mechanisms leading to delayed (secondary) loss of cells can be initiated, including the opening of connexin (Cx) hemichannels [25]. Following the latent phase is a phase of secondary deterioration, characterized by cerebral energy failure, seizures and cell swelling and ultimately substantial neuronal cell death [26,27]. Finally, there is a tertiary phase of injury in which there may be low levels of ongoing cell death due to loss of trophic support and altered connectivity [28,29,30].

The latent phase is the key window of opportunity to prevent the bulk cell death that occurs during the secondary phase. During this phase many processes are initiated that promote secondary cell death, including the opening of Cx hemichannels. Cx hemichannels are each half a gap junction, and can open to form large non-selective pores in the cell membrane. Evidence suggests that it is the opening of these channels that can lead to the propagation of inflammation and injury [31,32]. There is some evidence that gap junctions and pannexin channels could also be involved [32]. This review will discuss the evidence that gap junctions, Cxs, and pannexins play a role in perinatal brain injury and assess potential therapeutic strategies for perinatal brain injury that target these channels.

## 2. Connexin Proteins and HI

**Figure 1:**
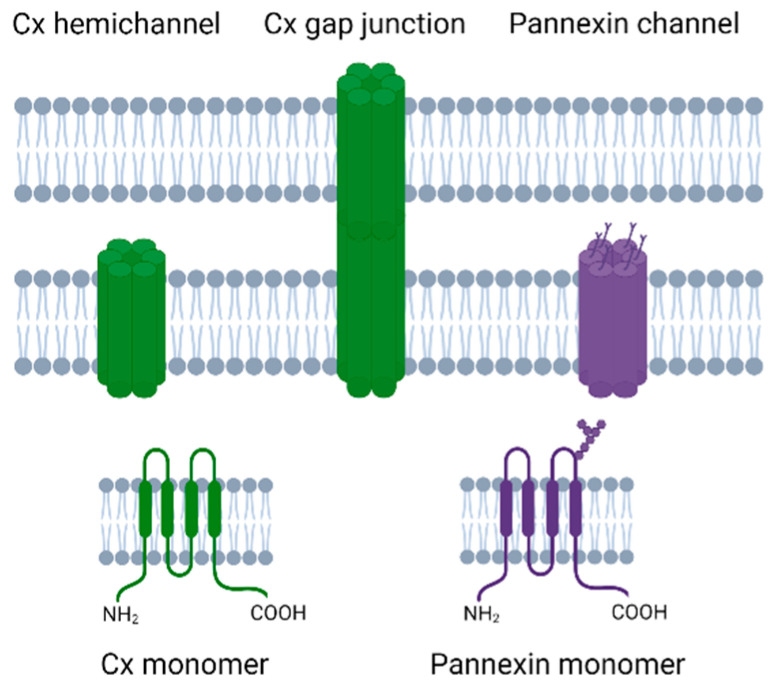
Schematic diagram showing the Cx hemichannel, gap junction and pannexin channels with the Cx and pannexin monomers. Cx hemichannels and pannexin channels can link the intracellular to extracellular environments whereas Cx gap junctions can link two adjacent cells.

Cxs are transmembrane proteins that have four transmembrane regions, an intracellular N-terminal and C-terminal, and two extracellular loops (Figure 1). Cx proteins can combine together as hexamers to form a Cx hemichannel, and two Cx hemichannels from adjacent cells can come together to form a gap junction [33]. Humans have 21 Cx proteins [34], of which Cx43 is of particular interest in perinatal brain injury as it is widely expressed in the developing brain including in astrocytes, microglia, and microvascular endothelium [35,36,37].

Cx43 protein expression has been shown to change in response to HI both in adult human brains after ischemic stroke at post-mortem [38,39], as well as in preclinical models of perinatal HI [40,41,42]. In near-term fetal sheep, there was significantly increased Cx43 mRNA levels in the intragyral white matter and cortex at 6 h after cerebral ischemia induced by 30 min of bilateral carotid artery occlusion [43]. In postnatal day (P) 7 rat pups significantly increased Cx43 protein levels were seen from 8 h to 7 days after HI induced by carotid artery ligation followed by inhalational hypoxia (the Rice–Vannucci model), compared with sham control [41]. However, another study in the same model showed a significant increase in Cx43 protein expression at 24 h, but not at 1 or 12 h, after HI in the subventricular zone compared with sham control animals [40]. In contrast, a study in P10 rats exposed to hypoxia alone (no ischemia) showed no significant difference in Cx43 expression in the hippocampus between those exposed to acute hypoxia (8 min 7% O_2_ followed by 4 min 6% O_2_, 2 min 5% O_2_, and 1 min in 4% O_2_) and controls at any time points between P10 and P45 [44]. This result in part could reflect the differences between HI and hypoxia alone.

It should be noted that Cx43 expression changes throughout the course of development [45]. For example, in fetal sheep there is increased Cx43 expression in the cerebral cortex in 0.9 gestation fetal sheep and newborn lambs compared with 0.6 gestation fetal sheep [46], and in rats there is increasing Cx43 expression from P0 to P28 in the cerebral cortex [47]. In fact, Cx43 can be detected in the ventricular zone of rats as early as embryonic day 14 [47]. This suggests that Cx43 is present from the very early stages of development, with increasing expression as development progresses.

Curiously, knockdown and knockout studies indicate that Cx43 has the potential to both potentiate as well as attenuate injury after HI. For example, reduced Cx43 expression in adult mice resulted in a larger infarct size and more apoptosis after middle cerebral artery (MCA) occlusion than in wild type mice exposed to the same procedure [48,49]. Further, knockout of just astrocytic Cx43 expression resulted in larger stroke volumes and increased apoptosis compared with mice with normal Cx43 expression after MCA occlusion [50]. In contrast, a more recent study suggested that knockout of astrocytic Cx43 expression increased protection of myelin integrity and reduced cognitive decline after chronic cerebral hypoperfusion induced by carotid stenosis in adult mice [51]. To further examine the role of Cx proteins one must separate the roles of Cx43 gap junctions and hemichannels.

## 3. Effect of HI on Gap Junctions

Gap junctions connect two adjacent cells allowing the movement of small molecules (<1.5 kDa) such as ions and second messengers between cells and play a key role in physiological conditions [52]. During HI, it appears that gap junctions remain open, for example, when Lucifer yellow is injected into astrocytes in rat brain slices there was staining in several surrounding cells, even in deoxygenated slice solutions, similar to that which occurs in normoxic conditions [53]. However, after HI it is suggested that gap junction intracellular communication (GJIC) decreases, for example, GJIC has been shown to decrease after 30 min of hypoxia in cultured rat astrocytes [54].

Whether this decrease in GJIC is neuroprotective or facilities the propagation of injury after HI remains to be determined. The ‘bystander death effect’ suggests a possible role for gap junctions in spreading injury between cells to previously uninjured cells, thereby exacerbating injury. Evidence for this includes the fact that blocking gap junctions with octanol 30 min prior to MCA occlusion reduced the infarct size in adult rats [55]. Furthermore injection of carbenoxolone immediately after intrauterine HI (induced through immersion of the intact uterus in saline) at 21 days gestation significantly reduced caspase-3 activation and reduced long-term neuronal loss in ischemic rat pups [56]. It should be noted, however, that these gap junction blockers are not specific to gap junctions only and some of this effect may also be through blocking Cx hemichannels. Elucidating the role of gap junctions alone from these studies is difficult.

The other possible involvement of gap junctions is that they could play a ‘good Samaritan ’role, and may spread toxic molecules to neighboring cells as a way to buffer toxic molecules to a non-toxic level, or they may spread ‘rescue messengers’ including adenosine triphosphate (ATP), reduced glutathione, and ascorbic acid to aid in cell survival [57]. Evidence for this includes blocking gap junctional coupling with carbenoxolone or 18α-glycyrrhetinic acid in mice astrocytic cell cultures, which resulted in increased glutamate cytotoxicity as measured by increased lactate dehydrogenase release [58]. Infusion of a mimetic peptide (Peptide5) at a dose of (50 µmol/kg/24 h) blocks Cx hemichannels only; however, at a higher dose of (50 µmol/kg/h) Peptide5 infusion blocks both Cx hemichannels and gap junctions [59]. In an experiment in a near-term fetal sheep, infusion of high dose Peptide5 90 min after ischemia resulted in a loss of electroencephalogram (EEG) recovery seen with the low dose peptide infusion and a greater increase in secondary impedance indicative of more cell swelling than the lower-dose peptide infusion [43].

Together these findings support the hypothesis that gap junctions may be involved in the development of perinatal brain injury, but whether they exacerbate or reduce injury likely depends on the stage of injury. Regardless, there is evidence that gap junctions may play a role in the propagation of injury, at least to immediately adjacent cells. However, the more far-reaching spread of injury is suggested to be due to the opening of Cx hemichannels [60].

## 4. Effect of HI on Connexin Hemichannels

Undocked Cx hemichannels are each half a gap junction and normally appear to remain closed [61]. However, Cx hemichannels can open during conditions that mimic ischemia and inflammation. For example, in cell culture, Cx43 hemichannels can open during conditions that mimic ischemia, such as oxygen-glucose deprivation (OGD), low extracellular calcium concentrations, and strong depolarization [60,62,63]. The molecular mechanisms resulting in the opening of these channels are still to be determined, but one possibility is that this occurs following s-nitrosylation of the intracellular Cx43 cysteine residue due to nitric oxide (NO) production that is said to increase hemichannel numbers in the membrane [64], although there is also evidence that NO can directly promote Cx43 opening through s-nitrosylation of cysteine 271 in *Xenopus* oocytes expressing Cx43 [65].

When these channels are open they form a large non-specific pore that can allow molecules of about 1 kDa in size to pass between the intracellular and extracellular environment [66]. The opening of these channels can lead to the influx of Na^+^, Cl^−^, and Ca^2+^ and the efflux of K^+^ ions, leading to cellular depolarization and cell lysis [67,68]. ATP, glutamate, and aspartate can also leave the cell leading to activation of purinergic signaling pathways and glutamate excitotoxicity [69,70,71]. Importantly, ATP release from hemichannels is an activator of signal 2 for the inflammasome (discussed later).

Unlike the role of gap junctions, there is extensive evidence to suggest that the opening of Cx hemichannels is directly involved in the propagation of injury and blockade of these channels is neuroprotective. For example in a Rice-Vannucci P7 rat model, pretreatment with Gap26, a mimetic peptide that blocks Cx43, 1 h prior to hypoxia significantly reduced infarct volume [41]. Delaying Gap26 treatment to 24 h after HI with daily injections for 7 days also resulted in significantly improved long-term neurological function. Multiple studies in near-term fetal sheep have also shown neuroprotection after blocking Cx hemichannels. Indeed, intracerebroventricular infusion of Peptide5 from 90 min until 25 h after global cerebral ischemia increased neuronal and oligodendrocyte survival 7 days after HI [43,72]. In the same model, Peptide5 blockade of Cx43 hemichannels has also been shown to improve survival of cortical GABAergic interneurons and prevented the loss of perineuronal nets 7 days after cerebral ischemia [73,74]. Furthermore, Peptide5 infusion has also been shown to improve EEG recovery and reduce seizure burden. Likewise, infusion of Peptide5 from 90 min until 25 h after complete umbilical cord occlusion in a preterm fetal sheep model has been shown to improve multiple outcomes, including survival of oligodendrocytes in the intragyral and periventricular white matter, survival of neurons in the caudate and putamen and recovery of EEG power and sleep state [75].

On the other hand, there was no neuroprotective benefit of blocking Cx43 hemichannels with Peptide5 if given before and during HI [72]. This suggests that these channels play a role in the downstream propagation of injury after the period of HI but do not significantly contribute to the development of injury during the insult. Although this does not resolve the precise duration that hemichannels remain open, the observation that a prolonged infusion with Peptide5 over 25 h provided greater neuroprotection than infusion for 1 h suggests that Cx hemichannels remain open for over 1 h after HI, reinforcing the concept that they are involved in the propagation of injury well after the initial insult [75]. Further, delaying the start of hemichannel blockade until 3 h after HI still reduced overall seizure burden, albeit with no significant effect on recovery of EEG power or histology [76]. This supports an ongoing effect after 3 h on propagation of electrical signals but suggests that starting intervention before then is critical to reduce acute injury.

## 5. Pannexin Channels

Pannexin channel proteins are more closely related to invertebrate gap junction proteins, called innexins, than to Cxs [77]. Pannexins have a similar topological structure to Cxs in that they have four transmembrane regions, two extracellular loops, and an intracellular N and C termini [73], but with a different homology [78,79]. Both pannexins and Cxs have traditionally been reported to form hexameric channel structures, although one recent report suggests pannexin channels may be heptameric [80]. Pannexins have N-glycosylation on the extracellular loop, which accounts for the fact that these channels generally cannot form cell–cell channels [81], albeit there are some suggestions that glycosylation of the pannexins can differ and allow cell–cell pannexin channels to occur [82,83]. There are three types of pannexins, two of which, Pannexin1 (Px1) and Pannexin2 (Px2) are highly expressed in the neonatal and adult brain [84,85]. These two pannexins differ in where they are predominately expressed with Px1 in the plasma membrane whereas Px2 is mainly expressed in the intracellular membranes [86].

Pannexin channels open in response to a range of stimuli including OGD, increases in extracellular K^+^, N-methyl-d-aspartate receptor (NMDAR) activation, and purinergic P2 receptor activation and reactive oxygen species [87,88,89,90,91]. Like Cx hemichannels, pannexin channels are permeable to molecules <1 kDa in size, including ions such as Ca^2+^, ATP, and glutamate [92,93]. Pannexin channel opening has also been shown to occur during anoxic depolarization ultimately leading to cell death [89,94].

## 6. Effect of HI on Pannexin Channels

The role of pannexin channels in the spread of perinatal brain injury remains unclear. However there have been multiple studies in adult models of ischemia that highlight a potential role for these channels. Blockade of Px1 with mefloquine injections has been shown to reduce infarct volume and improve motor scores post MCA occlusion in mice [95]. Also blockade of pannexin channels by intravenous probenecid administration prior to 20 min of global cerebral ischemia induced by common carotid artery occlusion reduced hippocampal neuronal death in rats [96]. It should be noted however that both these Px1 blockers are non-specific and they may also block Cx hemichannels, which may confound these results [97].

A study using Px1 and Px2 knockout mice showed that these mice have better functional outcomes and smaller infarcts after MCA occlusion than wild type mice [98]. A similar result was also observed in a Px1 knockout rat model in which there were significantly smaller infarct volumes in knockout female rats after MCA occlusion; interestingly this effect was not seen in male knockout rats [99]. Of note, there were increased cortical Px1 protein levels after ischemia in wild type females compared with males. The reason why females show greater ischemia induced activation of Px1 is unclear but it is suggested to be due to differences in estrogen receptor β signaling and caspase dependent cell death [99]. Whether or not this sex dependent effect of Px1 is observed in the neonatal brain is still to be determined.

Although pannexin channels can release ATP, they have also been shown to close upon high extracellular ATP concentrations. For example, when ATP is applied extracellularly, Px1 currents were inhibited in Px1-expressing oocytes [100]. This self-regulation of pannexin channels means that although these channels can open during ischemia and initiate injury, when ATP levels rise, they likely close. Indeed in the study described above by Wei et al., neuroprotection after MCA occlusion was the greatest when the Px1 blocker probenecid was given before reperfusion, than when delayed until 2 h after injury, and there was no neuroprotective effect when administration was delayed to 6 h [96]. This is consistent with studies of cell hypoxia–reperfusion injury where about one third of ATP release during ischemia was through pannexin channels (two thirds through Cx hemichannels), but all ATP release after reperfusion was through Cx hemichannels [101].

## 7. Inflammation in Perinatal Brain Injury

Perinatal brain injury due to HI or infection/inflammation results in an inflammatory response, which can result in long-term prolonged propagation of injury. This inflammatory response is part of the innate immune response. Inflammation is stimulated by either Danger Associated Molecular Patterns (DAMPs) or Pathogen Associated Molecular Patterns (PAMPs), which are recognized by pattern recognition receptors (PRRs) on immune cells [102]. The activation of PRRs is the priming signal 1 for inflammasome activation as the first step in the inflammatory response that results in the release of pro and anti-inflammatory cytokines and chemokines. Interestingly, Cx hemichannels release adenosine triphosphate (ATP) and whilst extracellular ATP has been reported to act as a DAMP, it is more importantly the key inflammasome signal 2 activator signal, triggering assembly of the inflammasome complex within the cell.

Elevation of pro-inflammatory cytokines is associated with adverse outcomes. For example, a higher blood level of interleukin (IL)-1, IL-6, IL-8 at the first or second day of life in a cohort of term infants exposed to perinatal asphyxia was associated with abnormal neurodevelopmental outcomes at 30 months of age [103]. In preterm infants the extremely low gestational age newborns (ELGANs) study showed that increased systemic levels of pro-inflammatory cytokines at one or more points between days 1 to 4 weeks of age are associated with adverse neurodevelopmental outcomes up to 10 years of age [104,105]. Given this evidence, there is substantial interest in determining the role of gap junctions, Cx hemichannels, and pannexins in potentially initiating but also propagating inflammation.

## 8. Astrocytes Involvement in Inflammation

Astrocytes are electrically non-excitable glial cells and are the most abundant neuroglial cell type in the central nervous system (CNS) [106]. Astrocytes highly express Cx43 but also Cx26, Cx30, Cx40, and Cx45 and many astrocytic functions rely on Cx protein expression [35,36,107]. Astrocytes form extensive connections between neurons and other astrocytes and propagate waves of Ca^2+^ through gap junctions [108]. Astrocytes have a critical role in maintaining homeostasis of the neural environment. This includes buffering extracellular K^+^ levels [109] and glutamate levels [110]. Astrocytes also can produce lactate from glycogen, which can be used by neurons to produce ATP [111].

After perinatal brain injury, astrocytic function can be compromised due to an increase in Cx hemichannel activity and a concurrent reduction in gap junctions, the latter leading to uncoupling of astrocytes [112]. Because Cx hemichannel-mediated ATP release activates the inflammasome, hemichannel opening results in the release of pro-inflammatory cytokines (including IL-1β and TNF) and other chemokines, and the release of ATP from astrocytes which can activate microglia, further potentiating inflammation [113]. Indeed exposing OGD/reperfusion injured microglial cultures to a medium from astrocytes that had also received OGD/reperfusion resulted in greater microglial activation and secondary pro-inflammatory cytokine release, which could be inhibited by blocking of Cx hemichannels with Gap-19 [114].

## 9. Inflammation and the Blood–Brain Barrier

The blood–brain barrier (BBB) is composed of capillary endothelial cells, capillary basal membrane, pericytes, and astrocyte end feet [115]. Cxs are expressed throughout these cells including Cx43, which is expressed on endothelial cells, pericytes, and astrocytes [35,36]; Px1 is also expressed on endothelial cells and astrocytes [116]. The BBB regulates the transport of nutrients, ions, and solutes into the CNS and the removal of toxic molecules back into the blood. Both Cx hemichannels and pannexin channels appear to have a role in physiological functioning of the BBB. For example, knockout of Cx43 and Cx30 in GFAP-positive cells has been shown to impair BBB integrity during increased hydrostatic vascular pressure and shear stress in mice [117].

It is well documented that after HI or infection the BBB becomes leaky, which exacerbates inflammation in the brain [118,119,120]. Both Cx hemichannels and pannexin channels have been implicated in this process [121]. The signal transduction pathways that lead to the increased permeability of the endothelial layer are complex but cytosolic Ca^2+^ increases have been shown to be involved [122]. For example, when bradykinin (an inflammatory peptide) was added to trigger Ca^2+^ oscillations, increased endothelial permeability was seen in bovine brain endothelial cells co-cultured with rat cortical glial cells [123]. These oscillations in Ca^2+^ could be inhibited by blockade of Cx hemichannels with carbenoxolone, Gap27, or with Cx43/37 knockdown. In addition, in vivo bradykinin administration resulted in leakage of dextran fluorescein into neural tissue but administration of bradykinin with Gap27, to inhibit Cx hemichannels, decreased the tissue leakage. Cx hemichannels and pannexin channels can also further regulate intracellular Ca^2+^ through ATP release as ATP can stimulate the release of Ca^2+^ from the endoplasmic reticulum [124]. This occurs through activation of purinergic receptors, which are found throughout the BBB including on astrocytes and endothelial cells and ultimately results in activation of the inflammatory paracrine pathway [121].

Cx hemichannels in particular have also been shown to be directly involved in the loss of endothelial cells in response to HI. For example, in a culture of rat brain microvascular endothelial cells exposed to hypoxia through flushing with 95% N_2_ and 5% CO_2_, cell loss was reduced through blocking Cx43 with a mimetic peptide [125].

## 10. Effect of Inflammation in the Absence of HI on Gap Junctions, Hemichannels and Pannexins

Exposure to inflammatory insults results, over time, in a decrease in overall Cx43 protein expression. For example IL-1β exposure decreases Cx43 protein expression in human fetal astrocytes [126,127] and daily intracerebroventricular infusions of lipopolysaccharide (LPS) into male adult rats decreased Cx43 protein expression in the hippocampus 14 days after the commencement of LPS administration [128]. Short-term exposure to IL-1β or TNF only led to changes in the phosphorylation state of Cx43, rather than decreased Cx43 expression at the transcriptional level [129,130]. However, phosphorylation levels critically regulate the open probability of both gap junctions and Cx hemichannels. Therefore, phosphorylation cannot automatically be assumed to result in an overall cumulative change in channel function. For example, Cx43 phosphorylation could contribute to an overall increase in GJIC even when overall Cx43 protein expression has been reduced. Interestingly, prenatal LPS injections to the mother did not change the total expression of Cx43 or Px1 in cultured astrocytes from offspring exposed to LPS compared to those of control astrocytes. However, compared with control conditions, the offspring of LPS exposed dams had increased Cx43 and Px1 expression in the cell membrane [131].

There is extensive evidence that exposure to inflammation in the absence of HI results in a decrease in GJIC [132]. Indeed exposure to inflammatory agents and cytokines such as LPS and IL-1β reduce GJIC in cultured astrocytes [126,133,134].

By contrast, Cx hemichannel activity actually increased after exposure to inflammation. For example in astrocytes co-cultured with microglia ethidium bromide uptake significantly increased after a 24 h treatment with LPS [135]. However, this effect was significantly blocked with the addition of Gap26 and Gap27 to inhibit Cx hemichannel activity. Furthermore, this effect was not detected in cultures of astrocytes from Cx43 knockout mice. Another study also showed increased activity of Cx hemichannels in acute brain slices previously exposed to *Staphylococcus aureus* infection in astrocytes bordering the abscess [136].

The effect of inflammation on pannexin channels is not as well understood. However, one study in mice showed that prenatal LPS exposure to the dam actually increased Px1 and Cx43 opening in cultured astrocytes from offspring [131]. Further, LPS exposure increased the opening of both Cx43 and Px1 ex vivo in acute brain slices in the adult offspring. The opening of these channels affected glutamate, intracellular calcium handing, and morphology of astrocytes and reduced neuronal survival [137], suggesting that both Cxs and pannexin channels remain open for periods after exposure to inflammatory cytokines. As noted above, though, the pannexin channel appears to be more involved in initial responses, while prolonged Cx hemichannel opening contributes to perpetuating the inflammation. Nevertheless, limited data suggest that cleavage by caspase-11 can transform Panx1 channels into a permanently open state, most likely in cells that are undergoing pyroptosis [138].

The reduction in GJIC and the increase in Cx hemichannel activity may be mediated through a p38 mitogen-activated protein kinase-dependent pathway [135]. This results in the expression of inducible nitric oxide synthase (iNOS) with a concomitant increase in NO production [139], which mediates S-nitrosylation of Cx43 and may increase channel numbers in the membrane. In turn, this may result in increased open hemichannel numbers under pathological conditions [64]. On the other hand, NO mediated S-nitrosylation of Px1 channels inhibits opening [140]. Therefore, these channels have been suggested to open via a different mechanism, as described in detail below [141].

## 11. Role of Connexins and Pannexin Channels in Propagation of Inflammation

Although inflammatory signals can cause the opening of Cx hemichannels and pannexins, the opening of these channels can further propagate inflammation. One of the key ways both Cxs and pannexins do this is through alterations in purinergic signaling due to the release of ATP. ATP is a crucial high-energy molecule in physiological conditions but under pathological conditions can act as a DAMP, triggering inflammation and exacerbating injury. When both Cx hemichannels and pannexin channels open, they can release ATP into the extracellular space. Indeed, after MCA occlusion, ATP levels in the extracellular fluid within the striatum have been shown to increase 2-fold from 3.1 nM to 5.9 nM 220 min after the occlusion [142]. However, currently extracellular ATP levels have not been examined in the developing brain [25].

Extracellular ATP can activate purinergic P2 receptors, including P2X receptors, which are cationic cell membrane channels. There are seven types of P2X subtypes, of which the P2X4 and P2X7 are of particular interest in the brain. Both of these subtypes are expressed on neurons and glial cells [143,144]. When these channels open they allow the passage of Na^+^, K^+^, and Ca^2+^ [145]. This results in an increase in intracellular Ca^2+^ that can trigger further opening of Cx hemichannels, further increasing ATP release in a positive feedback loop that is often called “ATP-induced ATP release” [146,147]. Pannexin channels can also release ATP in the initial stages, but through a complex negative feedback loop involving P2X7 receptors, pannexin channels close in a process known as “ATP induced suppression of ATP release” [148]. The major propagation of injury, and major player in chronic disease, therefore, appears to be the Cx hemichannel. It is of note that pannexins have also been associated with ATP-induced ATP release [149]. This may be a primary mechanism underlying intercellular propagation of Ca^2+^ signals [150]. Unfortunately, few studies have combined both pannexin channel and connexin hemichannel role analyses.

After HI there has been shown to be an increase in the expression of both P2X4 and P2X7 receptors. In hippocampal organotypic slices, increased P2X4R expression occurred 20 h after 3 h of OGD, which was partially prevented by the addition of the P2 antagonist suramin [151]. P2X4 protein expression has also been shown to be increased 4 h to 7 days after hypoxia (5% oxygen for 3.5 h) in P0 rats, compared with control animals [152]. Similarly, increased P2X4 expression was detected in the corpus callosum and cingulum one week after HI induced by carotid artery occlusion followed by hypoxia in P3 rats [153]. Further, de novo expression of P2X7 receptors in microglial cells was seen after MCA occlusion in rats [154]. P2X7 receptor expression has been shown to transiently increase 3-fold immediately after asphyxia, induced in P0 rats by placing the uterus in saline for 15 min [155].

Opening of both P2X4 and P2X7 receptors is involved in the activation of microglia and release of pro-inflammatory cytokines [25]. For example, inhibition of P2X4 with 5-BDBD in adult mice that had 60 min of MCA occlusion resulted in reduced infarct volume, reduced neurological deficit scores, and decreased levels of IL-1β 3 days post occlusion [156]. Furthermore, in this model, P2X4 receptor blockade significantly reduced microglial activation. In primary microglial cell culture, hypoxia (3% oxygen for 4 h) also resulted in an increase in TNF and IL-1β mRNA expression and microglia having a more rounded appearance, which was prevented by P2X4R blockade with trinitrophenyl (TNP)-ATP [152]. Evidence for P2X7 receptor involvement in microglial activation includes the fact that in adult rats the expression of P2X7 is co-localized with microglia 1 and 4 days after MCA occlusion [157]. However, knockout of P2X7R results in a significant reduction in microglial activation 72 after MCA occlusion compared with wild type mice [158].

## 12. Inflammasome Activation

**Figure 2:**
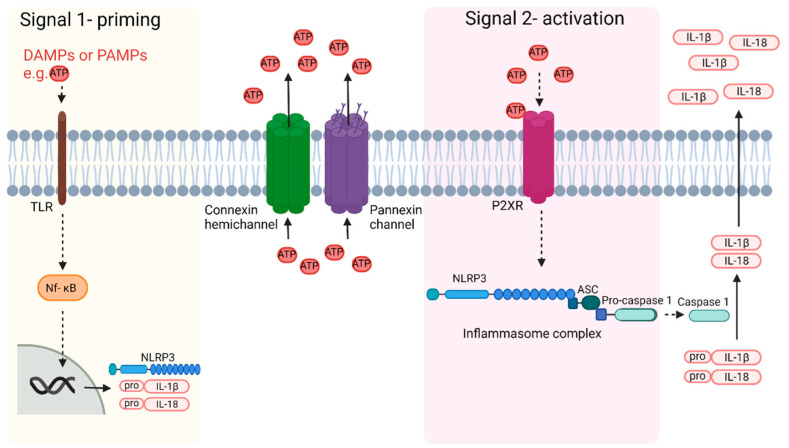
Schematic diagram of activation of the inflammasome. PAMPs and/or DAMPs act as the priming signal 1 and activate TLR, which triggers Nf-kB to move to the nucleus, ultimately resulting in the transcription of NLRP3 and pro-IL-1β and pro-IL-18. Opening of Cx and pannexin channels then results in ATP release. Extracellular ATP activates P2XR (and its breakdown products ADP and adenosine may act on their respective receptors) which acts as the inflammasome signal 2 activator, resulting in the assembly of the inflammasome complex. Caspase 1 cleavage of pro- IL-1β and pro-Il-18 then leads to the release of IL-1β and Il-18, which further potentiate the inflammatory process. Since the pannexin channel self-regulates in an ATP feedback loop, it is the Cx hemichannel which makes a major contribution during prolonged or perpetuated inflammation.

In addition to microglial activation, P2X7 receptor activation is also involved in the activation of the inflammasome (Figure 2). Inflammasomes are multimeric complexes of proteins, which are typically formed by apoptosis-associated speck-like protein containing a caspase recruitment domain (ASC), pro-caspase1 and a NOD-like receptors (NLR) family protein [159]. In relation to perinatal brain injury, one of the more characterized inflammasomes is the NOD-like receptor protein-3 (NLRP3) inflammasome [160].

There are two steps to NLRP3 inflammasome activation. Signal 1 is a priming step, which is induced by PAMPs including microbial toxins or DAMPs, or by endogenous inflammatory cytokines including TNF and Il-1β [161,162,163,164]. This leads to activation of the nuclear factor kappa-light-chain (Nf-kB) pathway which ultimately results in the expression of NLRP3, pro-IL-1β, and pro-IL-18 [165]. Signal 2 results in the activation of the inflammasome and is suggested to be induced by ATP, Ca^2+^ signaling, and K^+^ efflux, all of which, as mentioned previously, can be altered by Cx hemichannel activity [165]. Mitochondrial dysfunction, lysosomal rupture, and reactive oxygen species may also be activators although these are suggested to be downstream effects of inflammation rather than the initiators of inflammation [25]. Inflammasome activation leads to cleavage of pro-caspase 1 into caspase 1. Caspase 1 then cleaves pro-IL-1β and pro-IL-18 to IL-1β and IL-18. These can then be released from the cell through multiple mechanisms, as reviewed [166], as well as other cytokines. IL-1β can then induce neuronal injury through multiple mechanisms including through the induction of NO production, activation of apoptosis and programmed forms of necrosis (necroptosis), and modulation of the mitogen-activated protein kinase pathway (MAPK) [167,168].

There is increasing evidence that the inflammasome is activated after HI. For example, increased mRNA and protein expression of NLRP3, capsase-1 and IL-1β was seen 4–8 h after HI induced by carotid artery ligation followed by hypoxia in P7 rats [169]. Consistent with this, upregulation of NLRP3 in the hippocampus, striatum, and thalamus occurred 24 h after HI induced by carotid artery occlusion followed by hypoxia in P9 mice [170]. Moreover, in human neonates Il-1β and NLRP3 expression are altered during the first week of life and there is significantly higher expression of NLRP3 and ASC in school age children with neonatal encephalopathy (NE) compared with controls, suggesting that the NLRP3 inflammasome activation is involved in long-term persistent (chronic) inflammation [171].

Attenuating signal 1 or the priming step of inflammasome activation is not likely to be a viable therapeutic target due to the wide range of potential activators and the fact that this is likely to already have occurred by the time of diagnosis [172]. However, inhibiting the activation of the NLRP3 inflammasome by signal 2 could have therapeutic benefit with a more realistic window of opportunity for intervention. Indeed, ASC-deficient mice, exposed to hypoxia and carotid artery ligation at P9, had a smaller infarct size 7 days after HI in comparison with wild-types exposed to HI [170]. Interestingly, however, NLRP3-deficient P9 rats actually had a larger infarct volume 7 days after HI in comparison with wild-types exposed to HI [173]. This suggests that manipulation of ASC, rather than NLRP3 itself, may be a better target for therapeutic intervention. Another potential option would be to prevent inflammasome activation through the blockade of Cx43 hemichannels and potentially also pannexin channels. Indeed, a recent study showed that it is possible to reduce NLRP3 activation in a model of diabetic retinopathy through blockade of Cx43 [174]. Further potential therapeutic benefits of inhibiting these channels are discussed in greater detail below.

## 13. Therapeutic Potential of Connexin and Pannexin Blockers

Inhibiting Cx hemichannels and pannexin channels is a potential therapeutic strategy to reduce the progression of perinatal brain injury. However, some Cx hemichannel blockers such as carbenoxolone and glycyrrhetinic acid can be non-specific and can also inhibit gap junctions and pannexins [132]. Currently, there are three separate strategies that are Cx hemichannel specific: antisense oligodeoxynucleotides (although their prolonged use will ultimately reduce gap junction coupling), mimetic peptides [175], and small molecule hemichannel inhibitors such as tonabersat [101].

Antisense oligodeoxynucleotides (AsODNs) bind to specific mRNA and prevent the translation and consequently reduce the expression of the target gene, which may be of particular benefit given the upregulation of these genes as injury progresses [176]. Investigations into using particular Cx43 AsODNs in perinatal injury, to the best of our knowledge, has not occurred. Cx43 AsODNs have been shown to reduce inflammation and improve motor scores in adult rats 24 h after spinal cord injury [177] and to reduce inflammation and promote faster wound closure in a mechanical scrape wound model of corneal healing [178]. However, the therapeutic potential for Cx43 AsODNs is severely limited by the fact that they are rapidly broken down in the systemic circulation [179]. One option is to couple Cx43 AsODNs to a cell-penetrating peptide such as Xentry, which should increase the stability and bioavailability [180]. Indeed, in a study that coupled Cx43 AsODNs with Xentry, decreased Cx43 expression was seen in cultured ARPE-19 cells in both physiological and hypoxic conditions [181]. However, further studies in animal models are required to determine if this does indeed increase the stability and ability for systemic delivery.

Another option is mimetic peptides, which recognize channel-specific sequences and interfere directly with the desired channel [172]. Peptide5, for example, is hemichannel specific at appropriate doses and has been shown to block Cx43 [59]. As discussed above, Peptide5 has been used extensively in the fetal sheep models of HI brain injury and has been shown to have very promising neuroprotective effects [42,43,72,73,74]. However, the translational potential of this drug into clinical use is limited by its very short half-life.

Other extracellular loop peptides include Gap26 and Gap27 but, as reviewed elsewhere, these are known to also to block gap junctions [182]. Gap19 blocks the interaction between the carboxy tail and the cytoplasmic loop for Cx43 but does not affect gap junction functioning [183]. Indeed, Gap19 has been shown to reduce infarct volume, improve neurological scores, and reduce the in vivo concentrations of TNF and IL-1β after ischemia induced by MCA occlusion in adult mice [184]. Gap19 has poor cell penetration, which is critical as it needs to enter the cell to be active [183]. Previously Gap19 uptake has been improved using the TAT version of Gap19 [183,185]. However, there have been safety concerns regarding potential cytotoxicity [186,187]. Promisingly, a recent study that coupled Gap-19 with Xentry (Xentry-Gap19) showed increased GAP19 uptake into cells under both normal and hypoxic conditions [188], which may provide a novel approach to the administration of Gap19.

Tonabersat (also known as Xiflam) is a benzopyran derivate that can block Cx hemichannels [101]. Tonabersat has been investigated for a range of clinical purposes including for treatment of migraines and epilepsy and more recently in retinal eye disease [101,189]. Tonabersat has been used in controlled clinical trials and has been shown to have a high safety profile [190]. Importantly, tonabersat can also cross the blood–brain barrier [101,190,191]. It has proven effective in the treatment of migraine with aura [191], a form of migraine reported to be inflammasome mediated [192]. There are no studies that have reported the potential benefit of tonabersat in neonatal brain injury but with the potential to reduce seizures and the fact that tonabersat has recently been shown to reduce inflammation through inhibition of inflammasome activation, tonabersat may have potential as a neuroprotective treatment strategy [174,189,193]. Although tonabersat was originally reported to be a gap junction blocker, and has a moderate effect on cell–cell coupling in vitro [101,194], its main advantage is that it appears to be hemichannel specific in vivo. Further, it has a long half-life (enabling once daily dosing) and a well-characterized pharmacodynamic/pharmacokinetic profile. Extensive high dose toxicity and two-year carcinogenicity testing, and use with daily dosing in humans for up to 20 weeks [195,196] indicates that it cannot significantly uncouple gap junctions (as gap junction coupling is essential for organ function).

Currently there very limited studies investigating the potential therapeutic benefit of blockade of pannexin channels in perinatal brain injury. Two potential Px1 blockers are probenecid and Tenofovir. Probenecid was widely used for the treatment of gout [197] and has been shown to be protective against cerebral ischemia/reperfusion injury in adult rats [96]. However, to the best of our knowledge, there are currently no studies that have investigated the therapeutic benefit of pannexin blockade in relation to perinatal brain injury.

### 13.1. Add on to Therapeutic Hypothermia?

TH is the only established treatment strategy for moderate to severe HIE in term infants as it has been shown to significantly improve survival without disability and reduces severe disability [10]. However, in the RCTs, nearly half the infants treated with TH either died or survived with disability. Therefore, there is considerable interest to find combination treatments that can be used in addition to TH to further reduce brain damage and disability [20].

Currently there is only one preclinical study in a perinatal model that has combined the treatment of Cx hemichannel blockade with TH [76]. In this study, the combination of TH and infusion of Peptide5 began 3 h after global cerebral ischemia in near-term fetal sheep. Combination treatment was associated with a greater decrease in seizure burden compared with TH alone, but there was no additional effect on cell survival or EEG power. These results were surprising and suggest that there may be overlapping mechanisms of action between Cx hemichannel blockade and TH.

### 13.2. Treating Perinatal Brain Injury When TH Is Not Recommemded?

The greatest potential for Cx hemichannel blockade is its potential to be used as an alternative treatment to TH. TH has a high safety profile but has some limitations as noted above [198]. Critically, the recent HELIX trial showed that in low- and middle-income countries TH significantly increased mortality [18], likely because of a relatively higher proportion of injury evolving before birth [199].

TH is also not recommended for term infants with mild HIE due to the lack of RCTs directly investigating the potential therapeutic benefit of TH for these infants [200]. To the best of our knowledge, no studies have directly investigated the potential for Cx hemichannel blockade for the treatment of mild HIE. Given the evidence supporting the neuroprotective effects of Cx43 hemichannel blockade after severe HI in near-term fetal sheep, it is plausible that blockade of Cx43 hemichannels may also be an effective treatment for milder injury. Furthermore, the evolution of injury after a mild insult is likely to be slower and therefore there could be a longer therapeutic window of opportunity for successful intervention [15].

Finally, TH is not currently recommended for preterm infants with HIE. There is, however, evidence that Cx hemichannel blockade could be neuroprotective. Indeed in preterm fetal sheep, Peptide5 infusion started 90 min after 25 min of umbilical cord occlusion was associated with earlier recovery of EEG power and reduced neuronal loss in the caudate and putamen, although not in the hippocampus [75]. Further Peptide5 infusion was associated with increased survival of oligodendrocytes and improved recovery of oligodendrocyte maturation. This is a particularly important finding as it indicates that even in the very immature brain, Cx hemichannel opening contributes to the spread of injury.

## 14. Conclusions

Cx hemichannels appear to have a significant role in the initiation and propagation of perinatal brain injury. Pannexin channels have also been associated with at least initiation of ischemia induced pathology, although it remains to be proven if this is the case in the developing brain. Gap junctions may also have a role, but there is conflicting evidence for whether they exacerbate or attenuate perinatal brain injury. The prolonged propagation of injury and onset of chronic disease appear to be mediated primarily by Cx hemichannels, and Cx hemichannel blockade has ben shown to reduce acute brain injury in multiple animal models. Thus, there is potential for blockers of Cx hemichannels and pannexin channels to be developed into targeted interventions that could be used in conjunction with therapeutic hypothermia or as sole therapy.

## Data Availability

Not applicable.
